# Investigation of the Effect of PD-L1 Blockade on Triple Negative Breast Cancer Cells Using Fourier Transform Infrared Spectroscopy

**DOI:** 10.3390/vaccines7030109

**Published:** 2019-09-09

**Authors:** Mohamed H. M. Ali, Salman M Toor, Fazle Rakib, Raghvendra Mall, Ehsan Ullah, Kamal Mroue, Prasanna R. Kolatkar, Khalid Al-Saad, Eyad Elkord

**Affiliations:** 1 Diabetes Research Center, Qatar Biomedical Research Institute (QBRI), Hamad Bin Khalifa University (HBKU), Qatar Foundation (QF), P.O. Box 34110 Doha, Qatar; 2 Cancer Research Center, Qatar Biomedical Research Institute (QBRI), Hamad Bin Khalifa University (HBKU), Qatar Foundation (QF), P.O. Box 34110 Doha, Qatar; 3 Department of Chemistry and Earth Sciences, Qatar University (QU), P.O. Box 2713 Doha, Qatar; 4 Qatar Computing Research Institute, Hamad Bin Khalifa University (HBKU), Qatar Foundation (QF), P.O. Box 34110 Doha, Qatar; 5 Qatar Environment & Energy Research Institute (QEERI), Hamad Bin Khalifa University (HBKU), Qatar Foundation (QF), P.O. Box 34110 Doha, Qatar

**Keywords:** breast cancer, tumor cells, spectroscopy, FTIR, biochemical alterations, chemometric analysis

## Abstract

Interactions between programmed death-1 (PD-1) with its ligand PD-L1 on tumor cells can antagonize T cell responses. Inhibiting these interactions using immune checkpoint inhibitors has shown promise in cancer immunotherapy. MDA-MB-231 is a triple negative breast cancer cell line that expresses PD-L1. In this study, we investigated the biochemical changes in MDA-MB-231 cells following treatment with atezolizumab, a specific PD-L1 blocker. Our readouts were Fourier Transform Infrared (FTIR) spectroscopy and flow cytometric analyses. Chemometrical analysis, such as principal component analysis (PCA), was applied to delineate the spectral differences. We were able to identify the chemical alterations in both protein and lipid structure of the treated cells. We found that there was a shift from random coil and α-helical structure to β-sheet conformation of PD-L1 on tumor cells due to atezolizumab treatment, which could hinder binding with its receptors on immune cells, ensuring sustained T cell activation for potent immune responses. This work provides novel information about the effects of atezolizumab at molecular and cellular levels. FTIR bio-spectroscopy, in combination with chemometric analyses, may expedite research and offer new approaches for cancer immunology.

## 1. Introduction

Breast cancer is the most commonly diagnosed cancer in females worldwide, responsible for most cancer-related deaths among them [1]. The five-year survival rates from initial diagnosis for breast cancer patients have seen improvements in recent years, owing to better understanding of the development and progression of the disease. It is a heterogeneous disease, mainly categorized based on hormone receptor status; estrogen receptor (ER), progesterone receptor (PR) positive, human epidermal growth factor receptor 2 (HER2) positive, and triple negative breast cancer (TNBC) [2]. ER positive and HER2 positive breast tumors rely on estrogen and HER2 signaling pathways for survival and growth [3]. These pathways allowed therapeutic interventions via estrogen receptor antagonists such as tamoxifen, or drugs that impede the synthesis of estrogen such as aromatase inhibitors and HER2 inhibitors that include monoclonal antibodies and HER2 tyrosine kinase inhibitors [4]. TNBC is considered the most aggressive type of breast cancer which, although accounting for only 15–20% of all breast tumors, poses a significant increased risk of early metastasis within the first 3–5 years [5]. Moreover, systemic treatment choices are restricted due to the absence of expression of ER and HER2 [6].

Cancer immunotherapy has shown promising results in various human malignancies, with immune checkpoint (IC) inhibition at the forefront of successful immunotherapy regimes [7]. IC pathways attenuate T cell responses to prevent autoimmunity and maintain immune hemostasis. However, in the context of tumor immunity, interactions between ICs on T cells, and their ligands on antigen-presenting cells or tumor cells, provide significant barriers for tumor cell killing by cytotoxic T cells and assist tumor immune evasion mechanisms [7]. IC inhibitors target the interactions between immune cells and tumor cells. Inhibition of interactions between programmed death 1 (PD-1) with its ligand PD-L1 is one of the most prominent and effective IC inhibition strategies [7]. PD-1 has two known ligands, PD-L1 and PD-L2, which show different expression profiles on various tumors, with PD-L1 mainly being expressed on tumor cells [8]. MDA-MB-231 is a TNBC cell line that constitutively expresses PD-L1 [9]. Atezolizumab is a humanized immunoglobulin monoclonal antibody that selectively binds to PD-L1 to block PD-1/PD-L1 interactions, leading to sustained activation of tumor reactive T cells for potent anti-tumor responses [10]. Atezolizumab prolonged progression-free survival among TNBC patients as part of combination therapy with nab-paclitaxel [11], and recently received approval for the treatment of metastatic TNBC. Development of IC inhibitors involved series of preclinical and clinical trials to test the efficacy due to low response rates and immune-related adverse events in some cancer patients [7]. Therefore, better understanding of the interactions between IC inhibitors and their protein targets are warranted to develop robust predictive biomarkers for patient selection [7,12].

Fourier Transform Infrared (FTIR) spectroscopy is a potentially attractive diagnostic platform that can provide vital molecular and biochemical information [13]. Infrared (IR) spectrum originates from the vibrational motions of chemical bond atoms. When molecules absorb IR radiation, they produce bands at specific frequencies [14]. These bands yield detailed information about biochemical and chemical features at the cellular and/or sub-cellular levels [15]. Moreover, IR spectral bands are characteristic of proteins, lipids, cholesterols, phospholipids, carbohydrates, and nucleic acids [16]. One advantage of this technology is the exceptional sensitivity obtained at resolutions close to cellular levels; therefore, it can be utilized in the clinical field [17]. It is a simple, accurate, sensitive, highly-reproducible, and non-destructive technology for fast diagnosis in medicine and biological studies [18]. In contrast to standard histological staining methods, FTIR spectroscopy has the ability to simultaneously detect discrete changes in molecular structure and cell compositions. Direct biochemical analyses of all macromolecular components within cell samples can be obtained from a single data acquisition, without the addition of chemical stains or reagents and without disrupting the cells and tissue morphology [19].

FTIR spectroscopy has been used to screen and characterize a wide range of cells, tissues, and organs [20]. Importantly, it has been used to differentiate between normal, benign, and malignant tissues. Several studies have shown the application of multivariate statistical analysis in combination with FTIR spectroscopy to identify and differentiate between cancerous and noncancerous cells [21]. It has been applied to study different types of human and animal cancers such as breast [22], colon [23], prostate [24], brain [25], and cervical [26] cancer. Notably, FTIR analyses have been applied in breast tissue sections to investigate effects on extracellular matrix [27] and fibroblasts [28], to characterize different types of lymphocytes [29], and to distinguish most breast cancer cell lines grown in vitro after formalin-fixed paraffin-embedded (FFPE) tissue processing [30] or in spheroids [31]. FTIR has also been able to classify anticancer drug effects based on the drug-induced spectral perturbations observed on cancer cell lines [32]. Moreover, it has been used to analyze brain regions [33] and the pathological changes associated with stroke [34], traumatic brain injury (TBI) [35], and neurodegenerative diseases [36] such as Alzheimer’s disease (AD) [37], Parkinson’s disease (PD) [34], amyotrophic lateral sclerosis [38] and multiple sclerosis (MS) [39], cerebral malaria [40], epilepsy [41], and hemorrhagic stroke [42]. Furthermore, FTIR spectroscopy has also been applied to identify biomarkers in depressive disorder and monitoring the antidepressant effectiveness therapy [43]. In addition, FTIR spectroscopy has recently been applied for clinical *Candida* isolate identification and diagnosis [44]. Therefore, the multi-facet applications of FTIR analysis in various studies rationalize its utilization to investigate molecular changes in human cells in response to therapeutic modalities.

The aim of this study was to investigate the molecular and biochemical changes in MDA-MB-231 TNBC cells utilizing FTIR bio-spectroscopy after atezolizumab treatment. In addition, this study has shown the potential of FTIR to identify biomarkers through observed spectral differences, which could be potentially used to discriminate the atezolizumab-treated cells from the untreated cells. Immune checkpoint inhibitors have the potential to produce sustained tumor remission and induce potent anti-tumor immunity in breast cancer patients. Better understanding of the effects of IC inhibitors on tumor cells will assist favorable clinical outcomes.

## 2. Materials and Methods

### 2.1. Cell Culture

MDA-MB-231 breast cancer cell line (ATCC, USA) was maintained in RPMI-1640 medium supplemented with 10% fetal calf serum (FCS), 1% penicillin and streptomycin (Sigma Aldrich, St. Louis, MO, USA), and 1% Fungizone (HyClone, Logan City, UT, USA) at 37° C in 5% CO_2_. For IC inhibitor treatment, MDA-MB-231 cells were cultured on plates at a density of 2 × 10^6^ cells per 1 mL in the presence or absence of anti-PD-L1 monoclonal antibody (Atezolizumab, BioVision, Milpitas, CA, USA) at a concentration of 0.5 µg/mL, and incubated for 24 h in a humidified incubator at 37 °C and 5% CO_2_.

Three independent experiments of untreated (control) and treated MDA-MB-231 breast cancer cells were set up and five samples from each experiment were used for subsequent FTIR measurements.

### 2.2. Flow Cytometric Analysis

After treatment with atezolizumab, cells from treated and non-treated wells were trypsinized, washed, and re-suspended in 100 µL staining buffer (phosphate-buffered saline (PBS) with 2% FCS and 0.1% sodium azide) for surface staining. To gate out dead cells, 7AAD viability staining solution (eBioscience, San Diego, CA, USA) was used. PD-L1-Allophycocyanin (APC) (clone MIH1, eBioscience, San Diego, CA, USA) was then added and cells kept in 4 °C for 30 min. Cells were then washed twice with staining buffer and re-suspended in 300 µl for analyses. Data were acquired on BD LSRFortessa flow cytometer using BD FACSDiva software (BD Biosciences, San Jose, CA, USA) and analyzed on FlowJo version 10 software (BD Biosciences, San Jose, CA, USA).

### 2.3. Quantitative Real Time PCR (RT-qPCR)

Following treatment with atezolizumab, cells were collected from treated and non-treated wells to isolate RNA using an RNA/DNA/Protein Purification Plus Kit (Norgen Biotek Corp, Ontario, Canada) as per the manufacturer’s instructions. RNA from each sample was then reverse transcribed into cDNA using a QuantiTect Reverse Transcription Kit (Qiagen, Hilden, Germany). PCR reactions were performed on QuantStudio 7 Flex qPCR (Applied Biosystems, Foster City, CA, USA) using Fast SYBR Green Master Mix (Applied Biosystems, Foster City, CA, USA). All data were normalized to β-actin. Non-specific amplifications were checked by the use of melting curve and agarose gel electrophoresis. The relative changes in target gene expression were analyzed by using the 2-ΔΔCT method. The primers were designed using Primer3 software. The sequences of primers used are as follows;

- Human PD-L1 promoter forward, 5′-TGGCATTTGCTGAACGCATTT-3′.
- Human PD-L1 promoter reverse, 5′-TGCAGCCAGGTCTAATTGTTTT-3′.

### 2.4. Sample Preparation for FTIR Analysis

Following treatment with atezolizumab, cultured MDA-MB-231 cells were detached using 0.25% trypsin and EDTA (1 mM) for 3–5 min (all from Sigma, St. Louis, MO, USA) and washed thrice with NaCl solution (0.9%) to completely remove trypsin and culture medium. Cells were then resuspended in 1000 uL of 4% paraformaldehyde (PFA 4%) solution (Sigma, St. Louis, MO, USA) and placed on shaker for 30 min at room temperature. Cells were then centrifuged at 250× g for 5 min and washed thrice with sterile water to remove PFA. Approximately 1 × 10^6^ cells from treated and non-treated conditions were resuspended in 100 uL water and mounted on BaF_2_ disc (25 × 4 mm) (ThermoFisher Scientific, Waltham, MA, USA) and left to dry overnight. All cell lines were manipulated in exactly the same manner, allowing comparison of the spectroscopic features of the cell lines investigated.

### 2.5. FTIR Measurements

Fourier Transform Infrared (FTIR) spectroscopy spectra were recorded using a Nicolet iS50 FT-IR spectrophotometer in absorption mode within the range of 4000–1000 cm^−1^ with 64 scans per spectrum, 4 cm^−1^ spectral resolution. Background single beam spectra were measured on a substrate without biological cells by co-adding 128 scans. FTIR spectra were collected from three control and three treated independent cultures. For each culture, five samples were mounted on five different BaF_2_ discs and left to dry overnight. From each sample, 200 FTIR spectra were collected from different regions. Data processing, such as baseline correction and spectral average, were performed on the original FTIR spectra of each sample in order to perform the chemometric analysis.

### 2.6. FTIR Data Processing and Analysis

All data were generated using OMNIC™ Series Software, version 9 (ThermoFisher Scientific, Waltham, MA, USA). The spectra were processed using Origin software version 8. Spectra were acquired the cell layer sample away from the border between cell layer and a substrate to avoid resonance Mie scattering (commonly present in biological samples) [45]. FTIR spectra were vector normalized between 4000–1000 cm^−1^ and the baseline correction was performed on the full spectral range. Relative distribution of amide I protein components were determined from the second-derivative intensity spectrum. The relative amount of the different protein structures were quantified from the curve fitting of original IR spectra [46]. The contribution of different types of protein structures were calculated by curve fitting a linear mixed model of Gaussian bands [47]. Curve fitting was performed with MATLAB (version 2014a, MathWorks, Natick, MA, USA) over the spectral range 1700–1600 cm^−1^. For each cell culture, a mean and standard deviation was calculated and the average was generated. Mann–Whitney U test was performed on all data to test for significant differences. A *p*-value >0.05 was considered statistically non-significant. Previous studies have shown the detailed spectral band assignments of different biochemical content contributions [46]. Different biochemical make-ups were calculated from the area under the curve from specific bands (Supplementary Table S1).

### 2.7. Chemometric Analysis

Chemometric analyses, incorporating principal component analysis (PCA) and hierarchical cluster analysis (HCA), were performed to prove that the spectral differences measured between the control samples and samples treated with antibodies were significant. PCA allows for reduction of noise and capture of subtle differences within the spectral collection [48].

An IR spectrum composed of *p* = 6224 biologically meaningful wavenumbers within each spectrum in the spectral range of 4000 and 1000 cm^−1^ were initially used for PCA analysis. PCA generates *q* uncorrelated linear combinations of variables (referred as principal components [PC]) from the *p* × *p* covariance matrix *C*. Given a dataset *D* comprising of n_1_ spectra from control experiments and n_2_ spectra from treatment experiment, where each spectra has *p* wavenumbers, the covariance matrix *C* is defined as: *C = D^T^D*. We then performed a full eigen decomposition of the covariance matrix *C*, i.e., C×e=ƛ×e, where ƛ_i_ ∈ R and each e_i_ ∈ R*^p^*. Here, ƛ_i_ refers to eigenvalues which are used to estimate the amount of variance explained by their corresponding eigenvector e_i_. Each e_i_ ∈ e represents an orthogonal and uncorrelated principal component of the covariance matrix *C*. Usually, the maximum amount of variance in the data is captured by first *q* eigenvectors.

The infrared spectra were converted from Nicolet system format to comma separated values (CSV) to create data sets that were suitable for PCA analysis. PCA was performed using the “prcomp” function in the “stats” package in R [49]. In our experiments, we selected the top *q* = 3 PCs, as they capture approximately 100% of the variance in the data. We next used these eigenvectors to generate the loading vectors, i.e., f_i_ = $\sqrt{{ƛ}_{i}}\times$ e_i_, which effectively captures the wavenumbers corresponding to which their exists variations between the spectra of control and treatment experiments [50]. The primary results obtained from PCA are the score and the loading plots. The scores plots represent the spectra of these samples in a *q*-dimensional space of PCs. The loadings plot shows which wavenumbers are responsible in the data set for the maximum degree of separation inside this spectral collection.

The 3D scores plot of the PCs that explain the majority of the variance in the dataset enabled the spectra to be grouped according to the chemical information they contained [48]. Hierarchical cluster analysis was applied using the A2R package in R to compare the control and treated samples based on their PCA projections in the 3D score space [49]. HCA was used to group spectra that displayed the same degree of similarity by calculating the Euclidean distance between all the data sets using Ward’s algorithm. The result was visualized in a dendrogram and the grouping of the cells were presented as images consisting of color clusters according to the heterogeneity scale [49].

## 3. Results

### 3.1. Atezolizumab Effectively Blocks PD-L1 on Human Breast Cancer Cells

**Figure 1:**
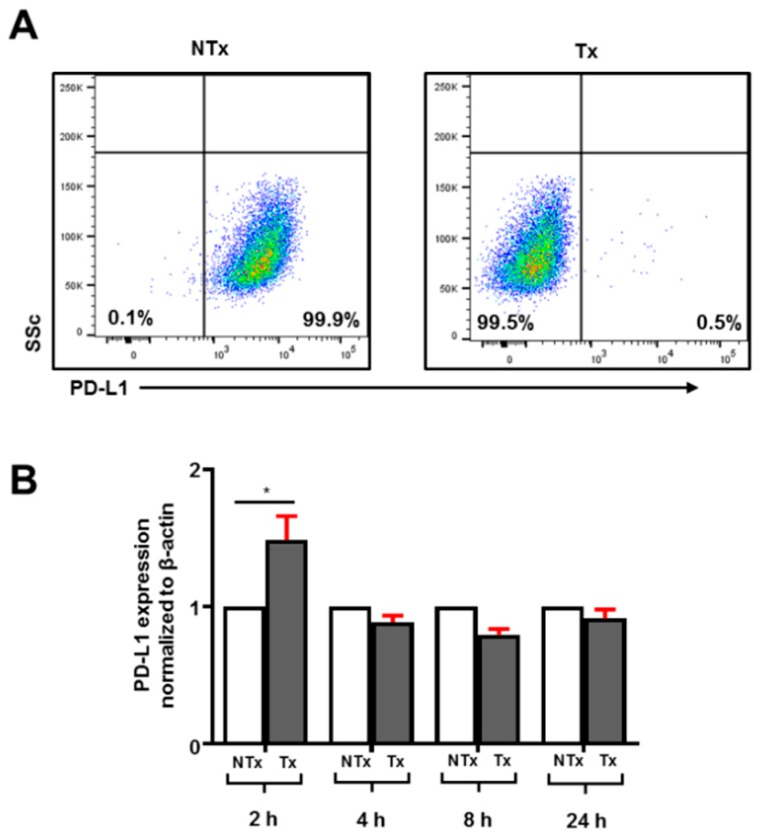
Effect of atezolizumab on PD-L1 expression on MDA-MB-231 cells. MDA-MB-231 cells were cultured in the presence or absence of atezolizumab for up to 24 h to investigate the effect of PD-L1 blockade on the surface and transcriptomic expression of PD-L1. Representative flow cytometric plots show PD-L1 expression on non-treated (NTx) and treated (Tx) MDA-MB-231 cells (**A**). The bar plot shows differences in PD-L1 transcriptomic expression in MDA-MB-231 cells up to 24 h Tx with atezolizumab (**B**).

PD-L1 expression has been observed on various tumors to assist escape from immune surveillance. Targeting PD-L1 is considered a plausible therapeutic approach in breast cancer patients. MDA-MB-231 are TNBC cells, previously shown to fully-express PD-L1 [9]. We also found that MDA-MB-231 cells are 100% positive for PD-L1 expression (Figure 1A). Treatment with atezolizumab for 24 h showed complete blockade of PD-L1 expression in MDA-MB-231 cells (Figure 1A). Interestingly, our transcriptomic analyses showed that atezolizumab does not affect PD-L1 mRNA expression (Figure 1B). In addition, previous studies have reported that certain drugs can have time-dependent effects on the protein expression at the transcriptomic level. Therefore, to find out if atezolizumab has any time-dependent effect on PD-L1 gene expression, we performed PD-L1 blockade kinetics for 2, 4, 8, and 24 h intervals and studied transcriptomic expression. We found that PD-L1 expression is increased initially following treatment with atezolizumab, but then returns to normal baseline levels (Figure 1B).

### 3.2. FTIR Spectroscopic Results

**Figure 2:**
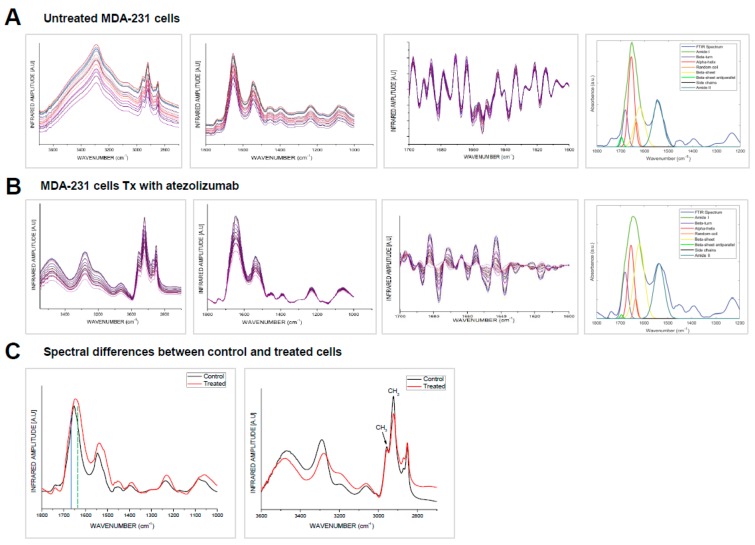
FTIR analysis of MDA-MB-231 untreated and treated cells with atezolizumab. FTIR analyses were performed on non-treated (NTx) and MDA-MB-231 cells treated (Tx) with atezolizumab after 24 h. Plots show FTIR spectra, second derivative and curve fitting of NTx MDA-MB-231 cells (**A**) and MDA-MB-231 cells Tx with atezolizumab (**B**). Plots show spectral differences between NTx and Tx MDA-MB-231 cells (**C**).

FTIR band assignments in this study were based on the specific spectral bands as defined in the Section 2 (Supplementary Table S1) [46,51,52]. We observed that spectral bands corresponding to lipid, protein, ester, nucleic acids, and carbohydrates dominate the FTIR spectra of the cellular structures (Figure 2). All samples were prepared, treated, and processed in the same manner.

We exploited for the first time, to our knowledge, the approach of IR-spectroscopy to study the secondary structure of the MDA-MB-231 cell proteins after treatment with anti-PD-L1 monoclonal antibody. We investigated IR absorption in the range of 1200–1800 cm^−1^, which is characteristic of amide band I, II, and III of proteins. The spectral differences showed that in the treated cells, there is little random-coil and α-helical content and predominantly β-sheet (Figure 2B), leading to a more stable and ordered protein relative to the untreated MDA-MB-231control.

Although amide I, II, and III spectral bands were used to study protein secondary structure, the main emphasis was on the amide I band. Amide I band mainly arises from backbone C=O stretching vibrations in the spectral range of 1700–1600 cm^−1^ [33,46]. The amide II band arises from backbone N-H bending and C-N stretching vibrations at 1580–1510 cm^−1^. The amide III band arises from C-N stretching, N-H bending, C-C stretching, and C=O bending at 1350–1200 cm^−1^. These two (amide II and amide III) bands have multiple assignment contributions, which makes the amide I band the most important and frequent band used to explain the secondary structure of polypeptides. The amide I band of treated cells’ spectra was found to vary in position, line shape, and intensity, which indicate biochemical changes in the cellular protein secondary structure. These spectroscopic changes were highly reproducible and were attributed to alterations in chemical compositions of the protein. Amide I band is composed of many contributions assigned to β-sheet within 1635–1610 cm^−1^; random coil at 1645–1630 cm^−1^; α-helical at 1660–1650 cm^−1^; antiparallel β-sheet and β-turn within 1695–1665 cm^−1^ (Supplementary Table S1) [46,51,52]. Positions of amide I components were determined from the second-derivative intensity spectra, while the relative amount of the protein structures were quantified from the original absorbance spectra (Figure 2A,B) [33,46]. The second-derivative spectra revealed that amide I band of untreated (control) cells was shifted from ≈1655 cm^−1^ (α-helical structure) to ≈1635 cm^−1^ (β-sheet conformation) in the treated cells.

IR spectra were collected from various regions of the untreated and treated samples in order to quantify IR absorption of protein components. The IR spectra of the control cells was dominated by amide I absorption in the range of 1600–1700 cm^−1^, with moderate absorption of amide II band at 1590–1560 cm^−1^. The spectra collected from various regions of the samples showed high similarity on position, amplitudes, and line shape. The IR spectra of the untreated cells showed that amide I band is the main contributor in the absorption and centered at ≈1655 cm^−1^, which arises from α-helical conformations (Figure 2A). Baseline correction and curve deconvolution of the average spectra provide an approximate and estimated protein content. The analysis showed that the amide I band of the untreated control cells was composed of α-helix at 1655 cm^−1^, β-sheet at ~1635 cm^−1^, and random coil at ~1630–1640 cm^−1^. The results showed that amide I components are overlapping and band ratio of α-helical/amide I composition was strong (0.58 ± 0.0825), β-sheet/amide I was moderate (0.34 ± 0.0456) and random coil/amide I moderate (0.12 ± 0.0084) (*p* < 0.05). The lipid ester band C=O at 1745 cm^−1^/amide I was moderate. The results also showed that the band ratio β-sheet/α-helical was moderate (0.59 ± 0.0531). The results indicate that the untreated control cells retain their native functional α-helical structure.

IR spectra have also been collected for the cells treated with the atezolizumab. The IR spectra are characterized by maxima at ≈1635 cm^−1^, which is characteristic for β-sheet conformations (Figure 2B). The curve deconvolution of the average IR spectrum of treated cells was composed of α-helical/amide I moderate (0.45 ± 0.0585), the β-sheet/amide I strong (0.52 ± 0.0728), random coil/amide I weak (0.08 ± 0.0024) (*p* = 0.0457). The lipid ester band C=O at 1745 cm/amide I was small. The results also reveal that β-sheet/α-helical was very strong (1.156 ± 0.104). Treated cells averaged spectrum in comparison to the untreated cells showed that there was a decrease in the α-helical and random coil contents, which was replaced by the β-sheet structure ≈1635 cm^−1^. These results reveal that untreated cells’ protein spectral band is dominated by α-helix structure, while the treated cells’ spectra were dominated by β-sheet conformation. This result indicates that the treated cells experience significant change in their protein biochemical make-up after atezolizumab treatment. The results reveal that there are no major changes in amide II and amide III due to treatment. In both cases, the results show that amide II/amide I was given by (0.567 ± 0.0396) and (0.563 ± 0.0401) for the control and treated, respectively, and amide III/amide I was given by (0.30 ± 0.015) and (0.38 ± 0.032) (*p* < 0.01) for control and treated, respectively.

The IR spectra of the untreated and treated samples show characteristic lipid bands at 2955 and 2922 cm^−1^ that assign for the lipid acyl (CH_2_) and lipid methyl (CH_3_) contents, respectively. The results show that the CH_3_/CH_2_ ratio in the untreated cells was much lower in comparison to the treated cells. This ratio reduction was also associated by decrease in the phospholipid band (C=O) at 1740 cm^−1^ in the treated cells. The IR spectra revealed that there is an increase in the olefinic = CH (unsaturated lipid) content at 3000–3027 cm^−1^ in the treated cells.

### 3.3. Chemometric Data Analysis

**Figure 3:**
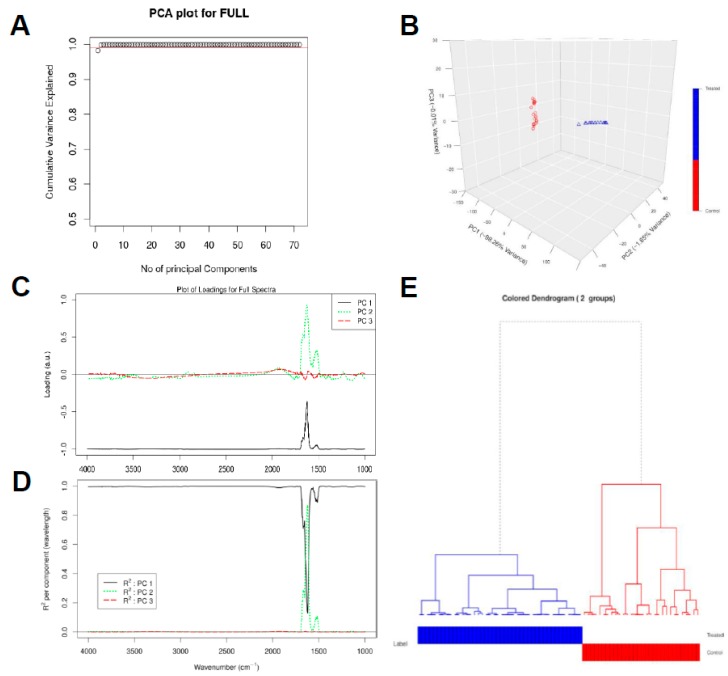
Chemometric analysis of FTIR experimental data on the full spectral range (4000–1000 cm^−1^). Amount of variance captured by PCs (**A**), PCA score plot (**B**), loading plots for the spectral data (**C**), squared correlation for the first three PCs for the spectral collection (**D**), and hierarchical clustering of the spectra for the different experimental samples (**E**).

As a further analysis of the FT-IR data, principal component analysis (PCA) was performed on the baselined collected spectra in the range of 4000–1000 cm^−1^. Figure 3A,B shows that the first PC captures more than 98.26% of the total variance in the data while the second and third PCs capture 1.65% and 0.01% of the total variance in the data, respectively. The PCA score and loading plots are able to extract unique fingerprint information about each cell type [33,46]. The loading corresponding to PC1 is negatively correlated with all the wavenumbers as observed in Figure 3C and can capture large variations (far from x-axis) between wavenumbers 4000–1700 cm^−1^ and 1500–1000 cm^−1^. PC1 helps to clearly distinguish the spectral samples belonging to the control experiment (negative values in PC1 dimension) from the spectra for the treatment experiments (positive values in PC1 dimension) as depicted in Figure 3B. The loading vector corresponding to PC2 can capture larger variations between wavenumbers 1750 cm^−1^ and 1500 cm^−1^, the region where PC1 can capture relatively less variance. Similarly, PC3 loading vector can capture small variations in the spectra between the wavenumbers 2000 cm^−1^ and 1750 cm^−1^.

Figure 3D shows that PC1 can explain the majority of the regions of the spectra considered during chemometric analysis. In particular, PC1 has high R^2^ and hence can explain well the regions between the wavenumbers 4000–1750 cm^−1^ and 1500–1000 cm^−1^. PC2 has a contribution in R^2^ in the wavenumbers between 1750 and 1500 cm^−1^. The sum of all the R^2^ value at each wavenumber should be equal to 1. Based on this principle, PC3 can explain very small variations in the regions between the wavenumbers 2000–1750 cm^−1^ as depicted by its contribution in the cumulative variance (Figure 3A). All other principal components would be associated with the noise in the spectral collection, as these 3 PCs together explain approximately 100% of the variance in the data (see Figure 3A,B).

The advantages of PCA are to allow the cells to be clearly distinguished into groups, allow for reduction of noise, and capture subtle differences within the spectra. Moreover, the PCA result makes it feasible to perform hierarchical clustering, thereby, combining untreated and treated cells using a dendrogram. A hierarchical dendrogram (HCA) was generated on all the spectral samples belonging to the untreated and the treated samples. For each cluster, we generated the representative low-dimensional embedding for that cluster by taking a mean over all the points in Figure 3B that belong to either the untreated or the treated samples, respectively. We then generated a dendrogram using the “A2Rplot” [53] function from the A2R package [53] in R. The resulting dendrogram is showcased in Figure 3E.

**Figure 4:**
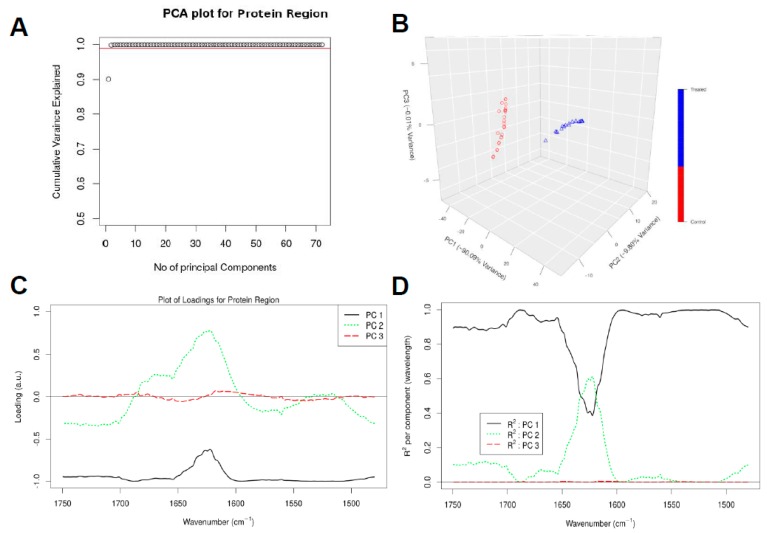
Chemometric analysis of FTIR experimental data on the protein region (1750–1480 cm^−1^). Amount of variance captured by PCs (**A**), PCA score plot (**B**), loading plots for the spectral data (**C**), and squared correlation for the first 3 PCs for the spectral collection (**D**).

In order to deeply investigate the conformation changes of the cellular protein, PCA analysis was performed only in the protein region of the spectra, i.e., we considered *p* = 623 wavenumbers in the spectral range of 1750–1480 cm^−1^ to see whether the spectrum belonging to untreated samples can be distinguished from those belonging to treated samples by focusing just in the protein region. We again used *q* = 3 principal components (PCs) as they capture approximately 99.9% of the variance in this spectral region (Figure 4A,B). The loading plots shows which wavenumbers are responsible in the data set for the maximum degree of separation inside this spectral region (Figure 4C).

Figure 4A, B shows that the first PC captures more than 90.09% of the total variance in the data, while the second and third PCs capture 9.80% and 0.01% of the total variance in the data, respectively. The loading corresponding to PC1 is again negatively correlated with all the wavenumbers as observed in Figure 4C, and can capture large variations between wavenumbers 1750–1650 cm^−1^ and 1600–1480 cm^−1^. PC1 again helps to clearly distinguish the spectral samples belonging to the untreated samples (negative values in PC1 dimension) from the spectra for the treated samples (positive values in PC1 dimension) as depicted in Figure 4B. The loading vector corresponding to PC2 can capture large variations between wavenumbers 1650 cm^−1^ and 1600 cm^−1^ in comparison to PC1.

From Figure 4D, we can observe that PC1 can explain majority of the regions of the spectra considered during chemometric analysis. In particular, PC1 has high R^2^ and hence can explain well the regions between the wavenumbers 1750–1600 cm^−1^ and 1600–1480 cm^−1^. PC2 has a contribution in R^2^ in the wavenumbers between 1650–1600 cm^−1^. All other principal components would be associated with the noise in the spectral collection, as these 3 PCs together explain approximately 99.9% of the variance in the data (Figure 4A,B).

**Figure 5:**
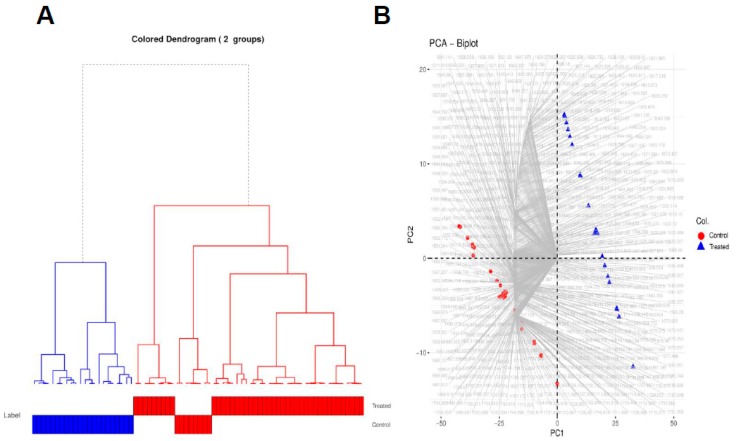
Hierarchical clustering of the spectral data of the samples. Hierarchical clustering of the spectra for the different experimental samples (**A**) and PCA biplot explaining which wavenumbers are associated with untreated versus treated samples (**B**).

We again performed a hierarchical clustering of all the spectral samples belonging to the untreated and treated samples, as illustrated in Figure 5A. For each cluster, we generated the representative low-dimensional embedding for that cluster by taking a mean over all the points in Figure 4B that belong to either the untreated or the treated samples, respectively. We can observe from Figure 5A that all samples belonging to control experiment cluster together and are discriminated from those belonging to treated samples.

We finally made a PCA biplot, which helps to provide information about which spectra regions are more correlated to which sample type and hence can better explain the differences between the untreated and treated samples. From Figure 5B, we can observe that majority of the spectra in the region 1650–1600 cm^−1^ are associated with the treated samples, suggesting that treatment with atezolizumab leads to formation of β-sheets in the proteins belonging to these samples. Similarly, the majority of the spectra in the region 1575–1550 cm^−1^ are primarily associated with the untreated samples.

## 4. Discussion

PD-L1 is frequently expressed in solid malignancies and the PD-1/PD-L1 pathway is exploited by various tumors to escape immune-surveillance. The constitutive expression of PD-L1 in tumors rationalizes the potential of targeting PD-L1. In this study, we investigated the effects of a PD-L1 inhibitor atezolizumab on its expression on breast cancer cells.

Studies have shown that tumors expressing IC ligands respond better to IC inhibitors, as PD-L1^+^ tumors were more responsive to PD-1/PD-L1 blockers compared to PD-L1^−^ tumors [54]. PD-L1 exerts immunosuppression by inhibiting CD8^+^ T cell cytotoxicity and enhancing immune evasion of tumor cells [55]. The responsiveness to anti-PD-1 therapy in tumors expressing PD-L1 is between 36 to 100%, whereas the responsiveness in PD-L1 negative tumors is up to 17% [56].

PD-L1 expression is suggested as a therapeutic target in TNBC [57]. It has been previously reported that targeting upstream signals via dual inhibition of STAT1 and STAT3 completely downregulates PD-L1 expression in breast cancer cells [9]. However, the exact mechanism of its expression regulation or mechanisms of blockade by therapeutic agents is not fully explored. We found that atezolizumab effectively blocked PD-L1 expression on MDA-MB-231 cells, as confirmed by our flow cytometric analyses. However, our transcriptomic investigations revealed that atezolizumab does not affect PD-L1 expression on mRNA level. Therefore, our results suggest that atezolizumab blocks signaling via PD-1/PD-L1 axis via some other mechanisms, not limited to only ‘masking’ the ligand. Thus, we investigated conformational changes in PD-L1 protein structure following treatment with atezolizumab.

FTIR bio-spectroscopy, combined with chemometric analysis techniques, were applied to delineate the biochemical alterations in the MDA-MB-231 cells after treatment with atezolizumab. A combination of FTIR and PCA analysis yielded novel results and revealed the biochemical changes that drive PD-L1 expression inhibition on breast cancer cells. Atezolizumab initiated biochemical alterations in lipid and protein contents of the treated samples. It caused protein conformation changes, as well as alterations in the lipid and phospholipid structures.

FTIR spectra of lipids were analyzed in the untreated and treated samples. We found significant alteration in the lipid acyl (CH_2_), methyl group (CH_3_), lipid ester (C=O), and olefinic = CH of the treated samples. An increase in the methyl group (CH_3_)/lipid acyl (CH_2_) ratio associated with reduction in the phospholipid content (C=O) might indicate to degradation of lipids into short chain fragments by oxidative stress. Oxidative stress and the resulting lipid peroxidation are responsible for the alteration of sub-cellular macromolecules [58,59]. Lipid peroxidation is associated with increased methyl (CH_3_) concentration and the formation of degradation products such as alkanes, carbonyl compounds, lipid aldehydes, and alkyl radicals [58,59,60,61]. Our results also show an increase in the olefinic = CH content in the treated cells, suggesting that cells might experience oxidative stress post-atezolizumab treatment.

Protein exposure to oxidative stress results in protein crosslinking, aggregation, fragmentation, and denaturation, resulting in loss of function. The levels of the aggregated protein were investigated from the amide I band at 1700–1600 cm^−1^ curve fitting. Interestingly, we observed that there is an increase in the band absorption of β-sheets at ≈1625–1635 cm^−1^ and associated with a decrease of α-helical absorption band at ≈1650–1660 cm^−1^, indicating protein aggregation and/or protein malfunction. This protein conformational change was not detected on the untreated cells, indicating that atezolizumab induces protein alterations and inhibits the protein expression. These results could not be obtained by other conventional biochemical assay techniques that could be used to determine the oxidative stress, which would be exceptionally difficult and inaccurate. Therefore, only the FTIR bio-spectroscopic approach that was used in this investigation is capable of revealing this level of biochemical details.

The chemometric analyses, PCA in the full spectral range of 4000–1000 cm^−1^, the protein region 1750–1480 cm^−1^, and the hierarchy dendrogram from HCA delineated a clear separation between the untreated and the treated samples. This clear separation reveals that the treated samples experience significant molecular changes in their biochemical makeup. One of these major bio-molecular alterations was found by curve fitting and PCA of the protein bands of the treated samples IR spectra. It revealed an increase in the β-sheet content and an associated with a decrease in α-helical secondary structure; again, the interpretation being that protein aggregation, a marker for sub-cellular alteration, occurred. The data also indicate that the treatment of breast cancer cells with atezolizumab can cause a much more global biochemical effect and is not only restricted to the effect of the sub-cellular protein.

Previously, FTIR spectroscopy has been shown to have a high potential to identify breast tumor tissue types [62]. Ali et al. managed to use two-dimensional (2D) correlation analysis in order to identify various cell types such as carcinoma cells, erythrocytes, lymphocytes, and the extracellular matrix (ECM) based on selected few wavenumbers. A simple model could be built using few wavenumbers to identify and discriminate between cells present in breast cancer tissue sections and the spectra of each type can be separated and clustered successfully [22]. We have also previously utilized FTIR spectroscopy as a bio-diagnostics tool for investigation of rat brain after ischemic stroke [46], characterization of different brain regions [33], and improving tissue classification [42]. We have also demonstrated the use of FTIR bio-spectroscopy, combined with another elemental analysis approach, to examine breast tissues in order to provide a single set of markers based on both organic molecules and inorganic trace elements [63]. Herein, for the first time, this study has explored the biochemical and molecular changes in breast cancer cells after atezolizumab treatment.

## 5. Conclusions

Our findings suggest that treating breast cancer cells with anti-PD-L1 monoclonal antibody (atezolizumab) might cause oxidative stress that can cause bio-molecular and sub-cellular alterations, which could be the major factor that affects the integrity, structure, and functionality of the of the treated cells. FTIR spectroscopy revealed that the biochemical and molecular alterations in the treated cells associated with this stress include: (a) A shift from random coil and α-helical structures to β-sheet conformation; (b) lipid peroxidation associated with oxidative stress; and (c) change in the phospholipid content. However, the alteration in the protein structure of the treated cells with atezolizumab could be used as a biomarker, as the treated cells also experienced much more global biochemical effect, which is not only restricted to the sub-cellular protein. This global effect might damage the cell receptors/ligands, as well as the cell membrane. These cascade effects lead to treated cells not able to bind to any other cells such as immune cells, blocking the PD-1/PD-L1 axis, which lead to sustained activation of immune cells with potent anti-tumor functionality.

Moreover, from the above conclusions, it is clear that our results show that FTIR bio-spectroscopy is a non-destructive, rapid, and refined technique to identify biochemical and sub-cellular changes in treated breast cancer cells. It can provide information about lipid degradation and protein structural changes. Usually, detection of these changes requires biochemical methods that include sample homogenization and/or treatment for immunoblotting or Immunohistochemistry (IHC), destroying the spatial–temporal integrity of the samples, which are not sensitive enough to detect these biochemical, molecular, and sub-cellular alterations. Therefore, we conclude that FTIR can complement and expedite research into cancer immunology.

## Supplementary Materials

The following are available online at https://www.mdpi.com/2076-393X/7/3/109/s1. Supplementary Table S1 lists the spectral assignments and protein components with their assigned wavelength in the IR region.
