# Supplementary material for: Investigation of the Effect of PD-L1 Blockade on Triple Negative Breast Cancer Cells Using Fourier Transform Infrared Spectroscopy
Source: Vaccines (Basel). 2019 Sep 9;7(3):109. doi: 10.3390/vaccines7030109 (PMC6789440; doi:10.3390/vaccines7030109)
Supplement: Supplementary file 1 [file vaccines-07-00109-s001.pdf]

Supplementary Table S1: The spectral assignments and protein components with their assigned wavelength in IR region

Infrared band	Integrated spectral range (cm^{-1})
**Lipid Components**	
**CH_2 symmetric stretching**	2852–2800
**CH_2 asymmetric stretching**	2915–2930
**CH_3 asymmetric stretching**	2950–2960
**∗C-H stretching**	2994–2800
**Olefin = CH**	3000–3027
**Carbonyl ester (C = O) stretching**	1745–1731
**Protein Components**	
**Amide I**	1700 – 1600
**Amide II**	1555 - 1535
**Amide III**	1350 - 1200
**Amide I Secondary structure (1700 – 1600)**	
**α-helix**	1660 - 1650
**Random coil**	1645 - 1630
**Parallel β-sheet**	1610 - 1635
**Beta-shoulder (1665 – 1695)**	
**β-turn**	1680
**Anti-parallel β-sheet**	1695

∗Total lipid regions.
